# Tobacco industry strategies undermine government tax policy: evidence from commercial data

**DOI:** 10.1136/tobaccocontrol-2017-053891

**Published:** 2017-10-09

**Authors:** Rosemary Hiscock, J Robert Branston, Ann McNeill, Sara C Hitchman, Timea R Partos, Anna B Gilmore

**Affiliations:** 1 Department for Health, University of Bath, Bath, UK; 2 UK Centre for Tobacco & Alcohol Studies, University of Nottingham, Nottingham, UK; 3 School of Management, University of Bath, Bath, UK; 4 Institute of Psychiatry, Psychology & Neuroscience, King’s College London, London, UK

**Keywords:** tobacco industry, price, taxation

## Abstract

**Objective:**

Taxation equitably reduces smoking, the leading cause of health inequalities. The tobacco industry (TI) can, however, undermine the public health gains realised from tobacco taxation through its pricing strategies. This study aims to examine contemporary TI pricing strategies in the UK and implications for tobacco tax policy.

**Design:**

Review of commercial literature and longitudinal analysis of tobacco sales and price data.

**Setting:**

A high-income country with comprehensive tobacco control policies and high tobacco taxes (UK).

**Participants:**

2009 to 2015 Nielsen Scantrak electronic point of sale systems data.

**Main outcome measures:**

Tobacco segmentation; monthly prices, sales volumes of and net revenue from roll-your-own (RYO) and factory-made (FM) cigarettes by segment; use of price-marking and pack sizes.

**Results:**

The literature review and sales data concurred that both RYO and FM cigarettes were segmented by price. Despite regular tax increases, average real prices for the cheapest FM and RYO segments remained steady from 2013 while volumes grew. Low prices were maintained through reductions in the size of packs and price-marking. Each year, at the point the budget is implemented, the TI drops its revenue by up to 18 pence per pack, absorbing the tax increases (undershifting). Undershifting is most marked for the cheapest segments.

**Conclusions:**

The TI currently uses a variety of strategies to keep tobacco cheap. The implementation of standardised packaging will prevent small pack sizes and price-marking but further changes in tax policy are needed to minimise the TI’s attempts to prevent sudden price increases.

## Introduction

Tobacco tax increases are the most effective and inexpensive way of reducing tobacco smoking prevalence,^1–9^ consumption,^10 11^ initiation^12 13^ and inequalities in smoking.^14–17^ Previous work revealed that, between 2000 and 2009 in the UK, the tobacco industry (TI) differentially shifted tax increases—absorbing tax increases on the cheapest cigarettes to keep them cheap (undershifting) while overshifting taxes on more expensive cigarettes to maximise profits.^18–20^ Consequently, the price of the lowest priced brands remained steady in real terms and the range between the cheapest and most expensive cigarettes increased. Similar TI pricing strategies have now been confirmed in other jurisdictions^21–23^ implying that this is an area of global concern.

Tobacco companies have been introducing increasing numbers of lower priced products^21–23^and more smokers, particularly the poorest,^9 24^ are now smoking these cheaper factory-made (FM)^21 25–27^ and roll-your-own (RYO) cigarettes.^9 25–27^ Evidence indicates that the availability of cheap tobacco reduces motivation to quit and quit success.^28–30^ This strategy is therefore likely to be driving inequalities in smoking and negating the intended benefits of tobacco taxation.^18–20^

### Objectives

Our previous 2000–2009 study^18–20^ examined only packs of 20 FM cigarettes using bi-annual data. Nothing is yet published on RYO price segmentation or what other pricing approaches the TI is now using. Little is also known about how prices vary through the year in response to tax increases. This study therefore aims to address these gaps by analysing commercial literature, monthly price sales and tax data to determine the strategies the TI is currently using to keep FM and RYO tobacco cheap including the extent and patterns of undershifting. Given the lack of research in this area and growing rates of RYO use in diverse jurisdictions, the results will be globally relevant.

### Background

In the UK, three types of tax are applied to tobacco: specific tax (a fixed amount per 1000 cigarettes or 1000 g of RYO tobacco), ad valorem tax (a proportion of the retail price) and value added tax (VAT, another ad valorem tax applied to most goods and services). Compared with ad valorem taxes, specific taxes tend to narrow the price range between premium and value brands, maximise the impact of tobacco taxes^31–33^ and raise more revenue.^34^ Since our previous project, UK tobacco taxes have increased annually, their structure has changed somewhat and the rate of VAT has varied (table 1). Most notably, since March 2010, tax increases between 1% and 5% above inflation have been specified each year for all tobacco products.^35^ In 2011, the UK increased the relative contribution of specific versus ad valorem tax for FM cigarettes^36^ and specified greater increases in taxes on RYO in an attempt to close the gap between FM and RYO prices. All tax changes were enacted annually each March with the exception of the 2009 change which took place in April.

**Table 1:** Tobacco-related tax changes and inflation during the study period

	Budget stipulated tax changes^81–87^			Tax rates^36 88^				Inflation: 12 month % changes*^89^	
	All tobacco	FM only	RYO only		FM		RYO		
				VAT†	Ad valorem	Specific	Specific		
				% pack price	% pack price	£ per 1000 sticks	£ per kg	CPI	RPI
Budget enactment									
22 April 2009	2% increase			15.0	24.0	114.31	124.45	2.3	−1.2
24 March 2010	1% above inflation‡			17.5	24.0	119.03	129.59	3.4	4.4
23 March 2011	2% above RPI	Ad valorem: 24%–16.5% Specific: 25% above RPI	+10% (total 12%) above RPI	20.0	16.5	154.95	151.90	4.0	5.3
21 March 2012	5% above RPI			20.0	16.5	167.41	164.11	3.5	3.6
20 March 2013	2% above RPI			20.0	16.5	176.22	172.74	2.8	3.3
19 March 2014	2% above RPI§			20.0	16.5	184.10	180.46	1.6	2.5
18 March 2015	2% above RPI			20.0	16.5	189.49	185.74	0.0	0.9

*Although the RPI (a measure of private household spending from survey data) is still employed in relation to tobacco taxation changes,^90^ it was de-designated as a national statistic in March 2013 (10) and replaced with the CPI which meets international standards and is comparable with other European countries.^91^ The RPI, unlike the CPI, includes housing costs, is more volatile than the CPI and rises more quickly.^92^

†VAT (a sales tax) changes did not occur on budget days.^88^ Changes occurred as follows: 01.01.2010 (17.5%), 04.01.2011 (20%).

‡2% above inflation for next 4 years.

§2% above inflation until end of next parliament.

CPI, Consumer Price Index; FM, factory made; RPI, Retail Price Index; RYO, roll your own; VAT, value added tax.

## Methods

### Review of commercial literature

A review of the commercial literature covering 2008–2014 was undertaken in order to explore how the TI was pricing its products, including the price segmentation used and whether other approaches were used to keep tobacco cheap or alter its price. Mentions of tobacco pricing and segments between 2008 and 2014 were collated from: tobacco company annual reports and presentations to investors; financial analyst reports; market reports (Euromonitor, Mintel, Keynote) and trade magazines (Retail News, The Grocer, Wholesale News and Tobacco Journal International), plus relevant articles from Forecourt Trader, Convenience store and Asian Trader (2014 only) collated for a related literature review. Finally, the segment descriptors identified in the literature were used as search terms in a Google.co.uk search.

### Sales and price data

Monthly Nielsen Scantrak data from January 2009 to December 2015 for the UK were used. Such data are collected every time an electronic barcode of FM cigarettes, RYO and make your own (MYO, where smokers own a machine which assembles cigarettes) tobacco is scanned via electronic point of sale system^37^ during purchase at a sales point in a participating retailer. Nielsen collect sales data from 87% of Great Britain’s supermarkets, 15% of its convenience stores (including 83% supermarket-owned convenience stores, 59% forecourts, 6% convenience store chains and 4% independents) and 17% of Northern Ireland stores with grocery sales (Northern Ireland represents 2.8% of the UK population^38^). This includes 100% of the big four UK supermarkets (Tesco, Sainsbury, Asda and Morrison). For other stores, stratified random sampling with replacement is used.^39^ These Scantrak data are then modelled so that they are representative of the UK using expansion factors based on the region, shop type and shop company.^39^

Like other products, tobacco brands can be arranged within a hierarchical architecture (see online supplementary table S1). Drawing on existing hierarchies,^40–42^ we used the following four-level hierarchy: brand (eg, Marlboro), brand family (eg, Marlboro Bright Leaf), brand variant (eg, Marlboro Bright Leaf Platinum) and, at the lowest level, stock keeping unit (SKU) (eg, Marlboro Bright Leaf Platinum 10 stick pack, single pack, price marked). Nielsen data were provided at SKU level, each SKU identifiable via an individual bar code.

10.1136/tobaccocontrol-2017-053891.supp1 Supplementary file 1

For each SKU, Nielsen identifies the brand family and variant, the number of sticks per pack for FM and grams per pack for RYO, whether sold in a single or multipack and (from August 2011) whether sold in a price marked pack. Data available for each month included: the total value of sales per SKU, the price per SKU, the number of SKUs sold, from August 2011 the number of FM sticks and kilos of RYO sold and from January 2013 the proportion of retailers to which each SKU was distributed. Data were analysed using SPSS V.22 and Microsoft Excel 2013.

### Segmentation

Consistent with our previous study,^20^ SKUs were allocated to price segments and price segment labels were identified based on the commercial literature review and an analysis of their relative pricing each month. Details are provided in online supplementary file 1.

### Analysis of pricing and volumes

Having allocated SKUs to segments, we explored prices, volumes, use of price-marking and variation in pack sizes by segment. In order to lessen small number anomalies, analyses were restricted to SKUs which reached a market share of >0.008% for at least 3 months (see online supplementary table S2).

#### Weighted average real prices

Weighted average real prices^20^ by segment were calculated by weighting for volumes sold and adjusting for inflation (thus creating real prices) using the Consumer Price Index (CPI).^43^ For each segment, we assessed pack and stick prices. For the latter, one RYO stick was set to contain 0.5 g tobacco based on the latest available survey data showing this is the average weight per RYO stick in England^44^ and the UK.^45^ For pack prices, preliminary analysis showed that pricing patterns over time for different pack sizes were similar, so data on all pack sizes were pooled to calculate weighted average real prices by segment for FM and RYO.

##### Impact of 2011 budget on RYO pricing

To understand whether the 2011 budget was successful in its objective of raising RYO prices, we compared the median monthly price rise in each RYO segment immediately after the 2011 budget enactment with median monthly price rises immediately following the other budget enactments and across the data series as a whole.

#### Volumes

As pack sizes changed over time, we assessed changes in sales volumes of FM and RYO over time using the number of sticks sold (again based on 0.5 g of tobacco per RYO stick). We assessed volumes in the tobacco market as a whole, for FM and RYO and by segment.

#### Pack size and price-marking

To examine changes in pack size over time, we identified popular pack sizes and calculated the market share by pack size for each segment annually over the study period. We explored patterns in the use of price-marking (printing the price on the pack, normally in bright bold font) by calculating the percentage of price marked packs by segment and pack size each month. We also calculated real weighted average prices (as above) and the % difference between the prices of price marked and non-price-marked packs in each segment.

#### Analysis of tax pass through

Tax pass through, the extent to which tax increases are passed through to consumers via increases in prices, or absorbed by companies through reduced profits, was evaluated through estimating changes in net revenue per pack. Net revenue is the money the company retains from its sales once all tobacco taxes and VAT have been paid. From this net revenue, companies cover their costs of production and distribution with the remainder being profits. As such, any tax rises not pushed onto consumers reduce net revenue and profits earned. The government specifies tax changes in the annual budget (which occurred in March in all of the study years except 2009 when it was April (table 1)). Tobacco tax changes were implemented at the time of the budget enactments but VAT changes occurred in January 2010 and January 2011.

The taxes for each pack (ad valorem, specific and VAT) were calculated and summed to deduce the total tax due (see online supplementary file 2 for methodology). This was then subtracted from the nominal pack price to estimate net revenue. Real net revenue was calculated by adjusting for inflation using the CPI and presented graphically by year and segment to examine patterns over time.

To examine the extent to which taxes were passed to consumers following each budget enactment and whether this varied by segment and the size of the tax increase, the net revenue per pack (in pence) for the month immediately prior to the tax increase was subtracted from the net revenue for the next month (up to the next budget) to estimate the change in net revenue. Changes for all pack sizes were graphed and showed similar patterns. We therefore only present changes in revenue for a popular pack sizes (20 s for FM premium and mid-price, 19 s for FM value and subvalue, and 12.5 g for RYO).

## Results

### Review of commercial literature

Growing demand for cheaper tobacco and the launch of additional cheap products (in both FM and RYO markets) were consistently reported during the study period.^46^ In 2012, Imperial Tobacco announced the creation of a new FM segment priced below existing segments, labelled ‘subvalue’.^47^ To encourage smokers to remain brand loyal,^48^ existing brands were extended with new SKUs introduced in lower priced FM segments^49^ or as RYO.^50^ New segmentation within RYO was also noted^51 52^ with RYO SKUs often introduced in lower priced segments.^53^

In addition to the creation of new, lower price segments, price-marking was used to sell selected SKUs at a cheaper price.^54^ This strategy was particularly noted for RYO^55^ and cheaper FM brands.^56^ Low pack prices were also maintained by cutting the amount sold per pack, for example from 20 to 19 sticks^57^ or launching smaller 10 g RYO packs.^58^ Other strategies included repricing brands from a high to a low segment,^53^ including papers and filters inside RYO tobacco packs (known as combipacks^59^) and launching MYO tobacco.^60^ Thus, more options were made available to smokers looking for cheap tobacco.

### Segmentation

Based on the commercial literature review and analysis of the price data, we created three segments for both FM and RYO: ‘premium’, ‘mid-price’ and ‘value’, with the addition, from 2012 onwards, of a fourth ‘subvalue’ segment in the FM market (see online supplementary table S3).

### Analysis of pricing and volumes

#### Weighted average real prices

##### Price per pack

**Figure 1:**
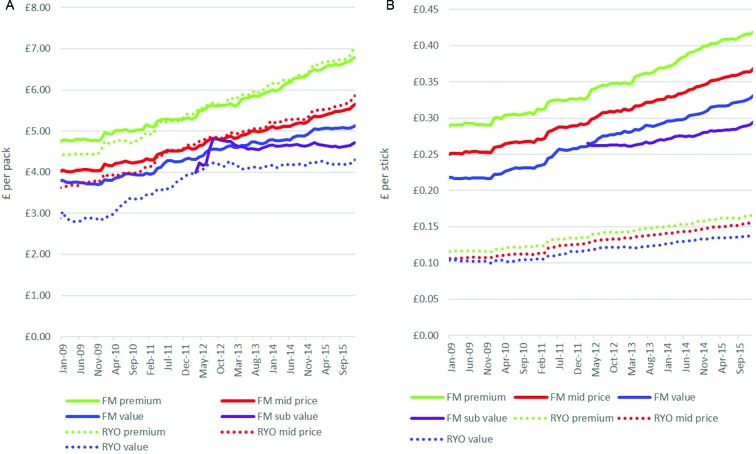
Real price per pack and stick for RYO and FM (single packs only-all pack sizes)*. *The price of subvalue packs appears unstable initially as different pack sizes were introduced. FM, factory made; RYO, roll your own.

When examining real pack prices, the prices of FM and RYO products in the premium and mid-price segments were similar (figure 1A). In contrast, the price of the value RYO packs was considerably lower than value FM packs. Around the time of the 2011 tax increase on RYO, the price per pack of RYO premium and mid-price segments began to follow the respective FM pack prices rather than being cheaper. The gap between value FM and RYO packs also narrowed at this time but had widened by the end of the data series.

Across the data series as a whole, the range in price per pack between the most and least expensive products within both FM and RYO increased. This is particularly so since 2012/2013 and is largely due to stagnation in the price of the cheapest products (figure 1A and online supplementary table S4). From January 2013 to December 2015, the real prices of the FM subvalue products actually fell 7 p and RYO value packs only increased by 2 p whereas the prices of premium FM and RYO products increased by £1.09 and £1.16, respectively.

##### Price per stick

When comparing prices by stick, RYO sticks were considerably cheaper than FM (figure 1B). The increasing price range within the FM and RYO markets also occurred for stick prices. For example, the price range between cheapest and most expensive RYO segment widened from 2 p to 3 p per stick and for FM widened from 7 p to 13 p. This reflected smaller price increases in the cheaper segments. However, price increases were seen in every segment suggesting the fall in price of subvalue packs is attributable to the declining number of sticks per pack.

Prices per stick also increased much faster among FM than RYO with the exception of subvalue FM. Thus, the range between the highest (FM premium) and lowest priced segments (RYO value) grew over time (£1.74 in January 2009 to £2.48 in December 2015). Nevertheless the gap between the cheapest FM and most expensive RYO changed little, again the result of the lack of price growth in the FM subvalue segment.

After the 2011 budget, when the emphasis of tobacco tax changed towards specific taxation, the gap between the FM premium and FM value segments declined from a high of 7.9 p per stick in January 2011 to 6.6 p in February 2012. After this point, the subvalue segment brands began to be introduced. Moreover, prices per stick of all three RYO segments rose by their highest amount after the 2011 budget enactment-premium rose 0.72 p, mid-price rose 0.65 p and value rose 0.42 p in April 2011 compared with median monthly rises of 0.03 p, 0.04 p and 0.03 p, respectively overall and median monthly post budget rises of 0.20 p, 0.19 p and 0.07 p, respectively in the rest of the data period).

#### Volumes

**Figure 2:**
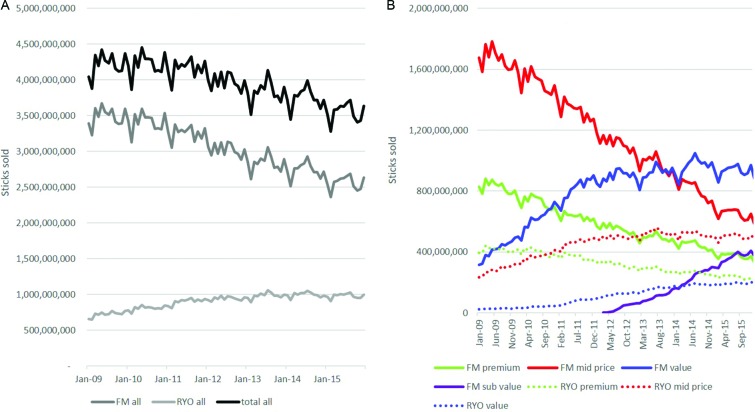
Volumes of sticks of FM sticks and RYO tobacco sold (A) overall and (B) by price segment. FM, factory made; RYO, roll your own.

Overall the total volume of FM and RYO sticks sold declined markedly in the UK: from 50.5 billion sticks in 2009 to 42.6 billion sticks in 2015 (a 13% decline) (figure 2A and online supplementary table S5). However, this overall decline consisted of a 17% decline in FM stick sales but a 46% increase for RYO sales, although RYO sales stabilised post-2012. Seasonal effects are apparent in both FM and RYO—New Year’s resolutions (and/or post-Christmas penury) impact tobacco sales.

Changes in volumes of FM and RYO sticks sold varied by segment (figure 2B and online supplementary table S6). Within FM, annual volumes of premium and mid-price products declined markedly throughout the period (54% and 61% declines, respectively). Volumes of FM value cigarettes grew by 126% over the study period although growth slowed from mid-2011. From their introduction in 2012, volumes of FM subvalue have grown to 4.3 billion sticks in 2015. Within RYO, from 2009 to 2015 premium sales declined by 43% while mid-price volumes grew by 78% overall although have been stagnant since 2013 and RYO value grew to 2.3 billion stick equivalents although growth slowed after 2012.

Thus, the market share of segments varied considerably over the study period. FM mid-price and RYO premium were the most popular of each type of product at the beginning of the study period whereas at the end of the study period FM value and RYO mid-price were most popular.

#### Pack size

##### FM

**Figure 3:**
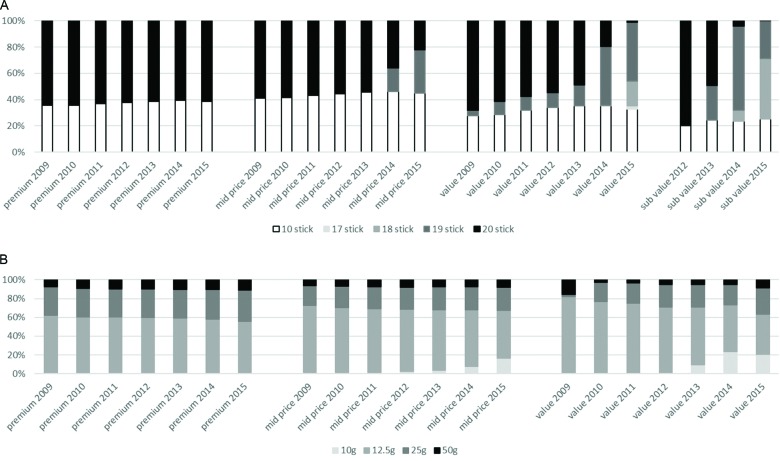
Market share of major FM and RYO pack sizes. FM, factory made; RYO, roll your own.

The variety of FM pack sizes increased over the study period most notably with the introduction of increasingly smaller pack sizes (17–19 stick packs) to an increasing number of brands (figure 3 and online supplementary table S7). Market share of traditional FM 20 stick packs declined in all segments but most markedly in the value and subvalue segments where, by 2015, only 1% and 0% of volume, respectively were 20-packs. By 2015, the pack size with the largest share was 18 sticks for the subvalue segment (46% share), 19 sticks for the value segment (45% share) and 10 sticks for the mid-price segment (45% share).

##### RYO

The most notable change in the RYO segment was the gradual advent of smaller, 10 g packs in recent years. This was seen most markedly in the mid-price and RYO value segments. For example, RYO mid-price 10 g packs increased from a 1% to 17% market share between 2009 and 2015. Throughout the study period, 12.5 g packs held the largest market share in all RYO segments but over time, this market share fell for all segments; for example, RYO premium 12.5 g packs declined from 62% market share in 2009 to 54% market share in 2015. Generally, the market share of largest pack size (50 g) grew slightly over the study period. For example, RYO premium 50 g packs increased from 8% market share in 2009 to 11% market share in 2015.

#### Price-marking

**Figure 4:**
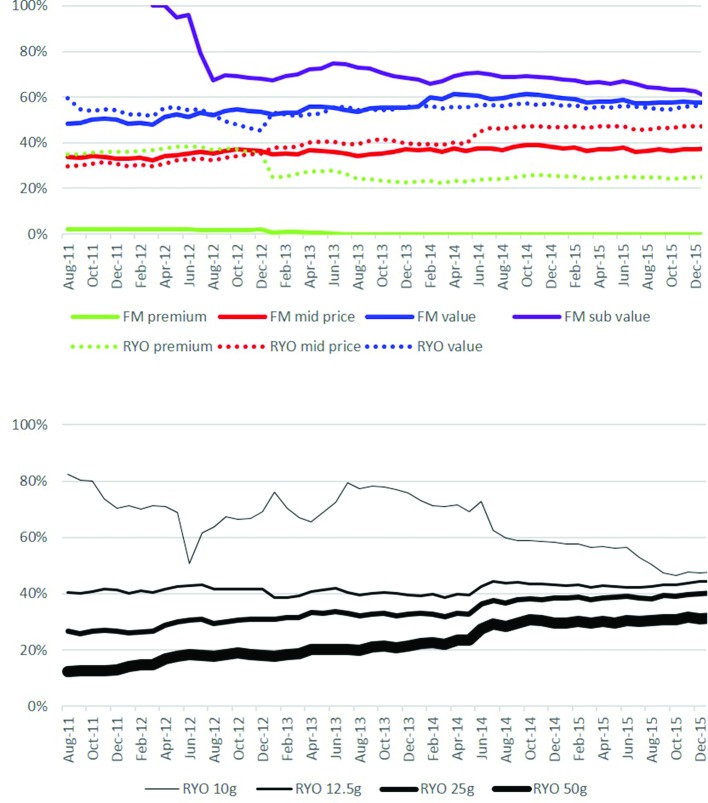
Percentage of packs price marked by (a) price segment and (b) pack size. Note: Nielsen data on price marking was only available from August 2011. FM, factory made; RYO, roll your own.

Cheaper segments were more likely to be price marked than expensive segments (figure 4A and online supplementary table S9A). For example, between 60% and 100% of subvalue FM products were price marked compared with a negligible proportion of premium FM products. Patterns for RYO products were similar although a higher proportion of premium RYO products were price marked (36% in 2012 and about 25% thereafter) than premium FM products. Half of RYO value products were price marked. Differences between prices of price marked and standard packs varied over time and between segments (see online supplementary table S10).

For RYO, but not FM, smaller packs were also more likely to be price marked than larger packs (figure 4B and online supplementary table S9). For most segments and pack sizes price-marking appeared to increase gradually over time. However, there were notable exceptions to this pattern. When the new subvalue FM brands were introduced in early 2012, 100% were price marked but this then fell rapidly to between 60% and 70%. There was substantial price-marking of 10 g RYO packs prior to growth in the mid-price segment (2011) and when 10 g packs were introduced into the value segment (2013) (figure 3B). In addition, price marking on premium brands in both FM and RYO segments has fallen.

### Tax pass through

**Figure 5:**
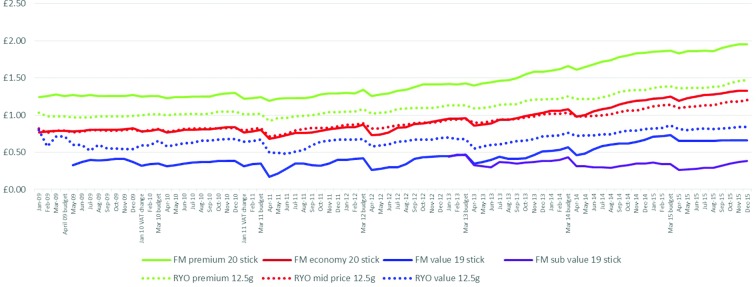
Net real revenue per pack by price segment for popular pack sizes. FM, factory made; RYO, roll your own.

Net real revenues were considerably greater for higher than lower priced segments although the gap was more marked for FM than RYO segments (figure 5 and online supplementary file table S11). A cyclical pattern of a drop in revenue immediately postbudget emerged with the 2011 budget and from this point there was progressively more differentiation in revenue between segments. Within the premium segment, revenue was greater on FM than RYO products while in the value segment, this pattern reversed and net revenues in the cheapest FM segment did increase throughout the whole period.

**Figure 6:**
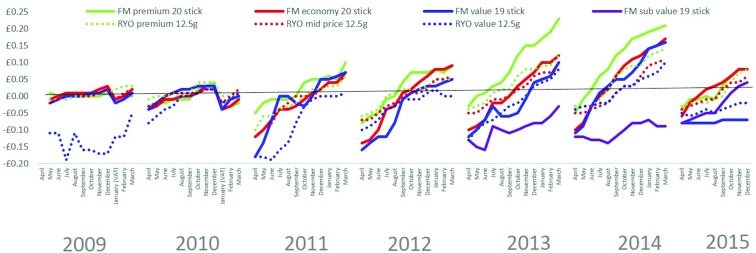
Change in net real revenue per pack postbudget (difference in revenue per pack in each postbudget month compared with budget month) by price segment for popular pack sizes. Note. A change <£0.00 indicates undershifting and a change >£0.00 indicates overshifting. FM value 20 stick pack shown for 2009 because 19 stick pack was not available at the time of the budget VAT changes in January 2009 and January 2010. FM, factory made; RYO, roll your own.

Declines in net revenue at times of tax (VAT or tobacco tax) increases can be seen more clearly using changes in net revenue postbudget (figure 6 and online supplementary file table S12). A decline in revenue postbudget indicates undershifting (ie, the industry has absorbed the tax increase through a decline in profit) while an increase indicates overshifting (ie, the industry has increased profits on top of the tax increase). The point at which the change equals zero is the point at which the tax is fully shifted to consumers.

Every year and in every segment, in the month after the budget, net revenues fell. Thus, tobacco tax changes were not passed straight onto consumers but were initially absorbed by tobacco companies (indicating slightly lower profit per pack). In general, the extent and duration of the undershifting was greatest in the lowest segments while in higher priced segments the undershifting was less marked and often short-lived with profits recovering to and then exceeded prebudget levels (indicating overshifting) within 1–3 months each year. For example, in 2014, revenue on premium FM products recovered to prebudget levels by May and by the end of the budget year was up by 23 pence. By contrast, revenues on subvalue FM products fell in 2014 until August, and though they increased thereafter, never recovered to prebudget levels.

Patterns varied somewhat by year reflecting the different tax changes. The years April to March 2009 and 2010 both saw VAT increases in January 2010 and January 2011, respectively. The declines in revenue at both these points indicate these VAT increases were absorbed by tobacco companies.

The years 2011 and 2012 saw the most marked tax changes—2011 saw a shift to, and significant increase in, specific duties and a marked increase in the tax on RYO, while March 2012 saw an increase in taxes of 5% above Retail Price Index (compared with 1% increase in 2010 and 2% increases in other years in the data series). In line with this, after the 2011 tax system changes, a more marked pattern of initial undershifting and greater differentiation in revenue change by segment emerged consistent with the widening revenue gap between segments.

## Discussion

This is the first study to provide a comprehensive overview of TI pricing including tax shifting throughout the year in both the FM and RYO markets. The commercial literature review and Nielsen data analysis both indicated that low priced FM and RYO tobacco products remained available throughout the study period via various pricing strategies including: launching new cheaper products; locking down the price of certain products through price-marking; introducing smaller packs and undershifting to ensure smaller price increases in the cheaper segments. Although real prices per stick have increased in all segments, real pack prices (and hence the price the consumer faces) of the cheapest FM and RYO products have remained static since 2012 largely due to the decline in pack sizes. Ten gram packs comprised a greater proportion of the cheapest RYO segments and were more likely to be price marked; thus the combination of small packs and price-marking appeared to be reinforcing. The price gap between FM and RYO was more complex: the gap in pack price appears to have narrowed between 2009 and 2012 particularly in the lowest segment (ie, between FM value and RYO value), signalling some success in using the 2011 tax changes to close the price gap. However, in terms of price per stick, the price gap increased across the dataset as a whole. Within RYO and FM, the range between cheapest and most expensive products has increased reflecting the differential shifting of taxes between segments. Hence, the opportunities for downtrading have continued to increase and volumes of the cheapest segments have grown in both FM and RYO.

TI pricing appears designed to deliberately undermine the public health impact of tax increases. Each year, immediately postbudget, the TI cuts its profits by absorbing the tax increases and thus preventing any sudden increase in price the consumer would face, instead smoothing that increase throughout the year. The degree and duration of undershifting is, in general, most marked in the cheapest segments and in recent years is more marked in FM than RYO. The industry then drives up profits later in the year with the extent of overshifting most marked in the more expensive price-segments. This approach to pricing means tobacco prices in the lowest segments are kept artificially low and leads to the growing gap in price between the most expensive and cheapest products.

Given that sudden large price rises are most associated with quit attempts,^28 61 62^ this pricing strategy and the consequent ongoing availability of cheap tobacco could reduce the incentive for price conscious smokers to quit.^28–30^ As such smokers are socioeconomically disadvantaged,^9^ this is likely to significantly exacerbate inequalities:^14–17^ UK data from October 2016 show smoking prevalence remains higher among the more disadvantaged (23.7%) compared with the more affluent (14.3%).^63^ As smoking is the leading cause of health inequalities^64^ this has significant implications.

### Limitations

Nielsen data are developed primarily for commercial purposes but are increasingly being used for academic research in several countries,^10 65–68^ and Nielsen tobacco data have been validated as having potential to enhance policy evaluation.^69^ Nielsen Scantrak data include only legitimate sales from UK grocery stores. However, such sales comprise up to 80% of the total market (legal and illicit) and illicit and non-grocery store sales are declining.^45 70^

The prices we used for RYO cigarettes include only tobacco, not papers or filters. Currently papers cost between 0.2 and 0.6 p each and filters between 0.2 and 0.8 p each,^71 72^ adding between 0.4 p and 1.4 p per cigarette.

We also note that price changes could (in part) have been caused by wider changes in the competitive landscape. This seems relatively unlikely: although there has been growth in e-cigarette sales, by 2015 the e-cigarette market had only reached a twentieth of the size of the tobacco market in the UK.^73^ Additionally, the tobacco market has remained dominated by the four Transnational Tobacco companies which collectively accounted for over 90% of the market, with only relatively small changes in their respective market shares.^74^

### Policy implications

Government tax authorities need to consider both the pack price confronting smokers in a retail environment and the price per stick. Prices (and profits) per stick can rise but by altering pack sizes, the industry can minimise the price rises consumers face. Minimal price increases were also occurring in segments with higher levels of price-marking. This paper therefore provides support for implementing standardised pack sizes of FM and RYO, banning price marked packs and other forms of price promotion. Standardised packaging legislation in the UK^75^ and the EU Tobacco Products Directive,^76^ both introduced in May 2016, preclude the use of price-marking and fix minimum pack sizes at 20 sticks FM and 30 g RYO. Our data show that in 2015 90% of packs within each RYO segment were under 30 g. As such these requirements will lead to marked changes in available pack sizes. In addition to analysis of the impacts of these changes, qualitative work may be needed to explore the impact of larger pack sizes on smokers attempting to quit, given that smokers wanting to quit prefer smaller packs.^77^

The industry’s ability to introduce new cheaper variants of existing products^40^ could be restricted by allowing only one variant per brand as is the case with new legislation from Uruguay^42 78^ and/or freezing the introduction of any new FM or RYO brands which could be part of an ‘End-game’ strategy.

The increase in tax on RYO in the 2011 budget had some success in narrowing the gap in the price between FM and RYO equivalent packs (despite the marked undershifting in the RYO value segment that year). However, a gap in price between product types still exists and is particularly marked when examining prices per stick. This and the fact that, within the value/subvalue segments, the degree of undershifting is now lower and profitability higher for RYO than FM, suggests there is scope for a further tax increase on RYO.

The marked price differentials within the RYO and FM markets which arise from the TI’s differential shifting of taxes between segments also need to be addressed. The minimum excise tax,^79 80^ implemented in May 2017 should prevent tobacco being sold below a price floor, and these results suggest that this or a minimum consumption tax (which includes VAT) should be considered elsewhere. Within FM, a further shift to specific taxes would help.

In addition, since the TI appears to be minimising sudden price rises which are more effective in stimulating quitting,^28 61 62^ the timing of tax increases could be made less predictable to make it harder for the industry to use pricing to undermine their public health impact. Furthermore, the government could stipulate only a couple of dates per year when price rises could be made to help ensure each tax increase is passed on in a timely manner and consider larger tax increases in anticipation of industry undershifting.

In summary, cheap tobacco has been made available in the UK through TI strategies of absorbing tax increases in the cheapest segments, introducing new cheaper products, price-marking and reducing pack size for both FM and RYO. New approaches are needed, such as fully specific taxation, tax increases on RYO, restricting brands to one variant and preventing the introduction of new brands. Internationally, a minimum excise or consumption tax and standardisation of packs (to include a minimum pack size or weight and prevent price marking) is recommended.

**What this paper adds**

- In the early 2000s, tobacco companies were making cheap cigarettes available in the UK through overshifting taxes on premium factory-made (FM) brands and undershifting taxes on cheap cigarette brands. When cheap tobacco is available smokers are less likely to quit smoking. Nothing is known about pricing of roll-your-own (RYO) tobacco.
- Tobacco companies take a similar approach to pricing RYO and FM with the price segmentation previously seen in FM now present in RYO. They are using a variety of strategies to ensure cheap tobacco remains available and to minimise the impact of tax changes: launching new cheaper products and smaller pack sizes, price-marking and undershifting taxes particularly in the cheaper price segments to smooth price rises consumers face and prevent sudden large increases. Consequently, price differences between the most expensive and cheapest products continue to grow both within and between FM and RYO, and volumes are only growing in the cheapest price segments of each. Policy changes that will help include: considering both pack and stick price in setting taxes and accounting for the correct weight of tobacco in a RYO cigarette; a wholly specific tax structure; increasing taxes on RYO to approximate those on cigarettes; restricting brands to one variant and preventing the introduction of new brands. Internationally, a minimum excise or consumption tax and standardisation of packs (to include a minimum pack size or weight and prevent price marking) is recommended.
