# Correction: *Tobacco industry strategies undermine government tax policy: evidence from commercial data*

**DOI:** 10.1136/tobaccocontrol-2017-053891corr1

**Published:** 2020-09-01

Hiscock R, Branston JR, McNeill A, *et al*. Tobacco industry strategies undermine government tax policy: evidence from commercial data. *Tobacco Control* 2018;27:488-497.

In the original article there was an error in the calculation of Value Added Tax (VAT). We calculated the VAT sales tax that is applicable to tobacco sales in the UK as a proportion of the final sales price rather than as a proportion of the pre-VAT price, leading to a slight over-estimation of the size of the tax and hence an underestimation of the industry’s revenues. For example, in December 2012 the average price for a pack of premium cigarettes was £6.85. We originally calculated the VAT liable to be £1.37 but the correct VAT liable was £1.14. Thus, the industry revenue was underestimated by about 23 p: originally the net revenue was calculated as £1.41 whereas the corrected net revenue was £1.64. This error does not change the fundamental result or substance of the paper. Thus, no text requires alteration.

The difference was greater for later months in the dataset and for more expensive brands as VAT is a tax on value (updated table 1 & figure 5 reproduced here). However, the percentage change was greater for cheaper brands as the change represented a higher proportion of a lower price. Similarly, when looking at updated and original change in net revenue post budget (updated and original figure 6 reproduced here), the recalculation made a greater difference further from the budget (average change from previous month April to October: 0 p, November and December: 1 p, January to March: 2 p). Thus,figure 6 the tobacco industry was able to overshift to a greater extent than thought previously, after initially undershifting at the time of the budget.

**Table 1:** Comparison of industry revenue as featured in the original paper against the corrected figures for the first and last month of the data series

	Jan-09				Dec-15			
	Original	Corrected	Under reporting	% Original	Original	Corrected	Under reporting	% original
FM premium 20 stick	£1.24	£1.36	-£0.12	10%	£1.95	£2.22	-£0.27	14%
FM economy 20 stick	£0.77	£0.86	-£0.09	12%	£1.33	£1.57	-£0.24	18%
FM value 20 stick	£0.38	£0.46	-£0.08	21%	£0.66	£0.85	-£0.19	29%
FM sub value 19 stick					£0.38	£0.57	-£0.19	50%
RYO premium 12.5g	£1.03	£1.09	-£0.06	6%	£1.47	£1.61	-£0.14	10%
RYO mid price 12.5g	£0.82	£0.87	-£0.05	6%	£1.20	£1.33	-£0.13	11%
RYO value 12.5g	£0.80	£0.86	-£0.06	7%	£0.84	£0.96	-£0.12	14%

**Figure 5:**
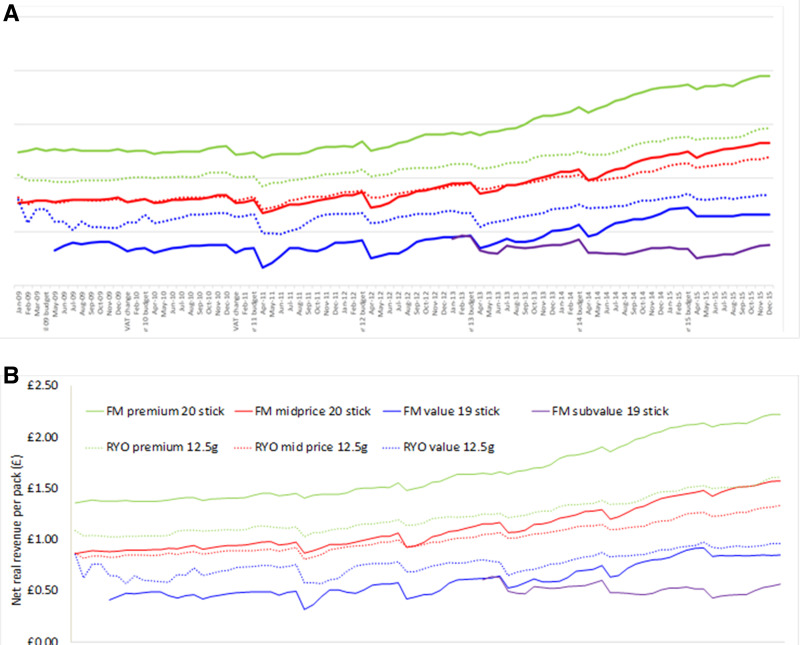
Net real revenue per pack by price segment for popular pack sizes. FM, factory made; RYO, roll your own (a) originally and (b) post VAT recalculation.

**Figure 6:**
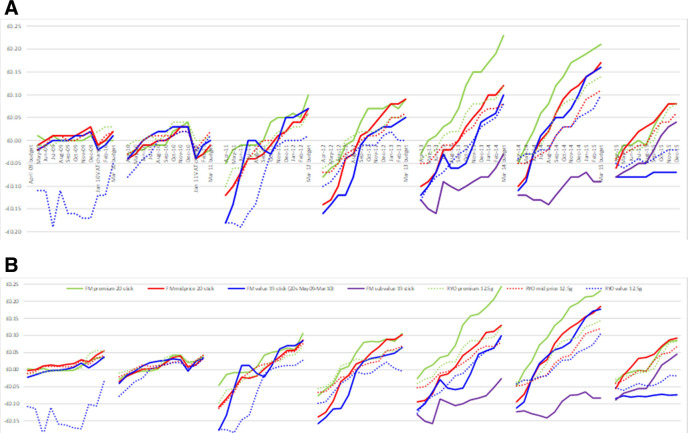
Change in net real revenue per pack postbudget (difference in revenue per pack in each postbudget month compared with budget month) by price segment for popular pack sizes for (A) original paper and (B) revised VAT. Note: In figure 6, a change <£0.00 indicates undershifting and a change >£0.00 indicates overshifting. FM value 20 stick pack shown for 2009 because 19 stick pack was not available at the time of the budget VAT changes in January 2009 and January 2010. FM, factory made; RYO, roll your own.

