# Supplementary material for: Tobacco industry strategies undermine government tax policy: evidence from commercial data
Source: Tob Control. 2017 Oct 9;27(5):488–97. doi: 10.1136/tobaccocontrol-2017-053891 (PMC6109235; doi:10.1136/tobaccocontrol-2017-053891)
Supplement: Supplementary file 1 [file tobaccocontrol-2017-053891supp001.pdf]

Supplemental file: Tobacco industry strategies undermine government tax policy: evidence from commercial data

Supplemental file 1 Details of segmentation development

To help understand the paper and the segmentation process we provide terminology on brand classification (Table S1).

Table S1 Comparison of hierarchical brand classification typologies

	Generic brand Hierarchy e.g. motor car[1*]	Philip Morris International (global) [2]	Australia[**]	Nielsen (UK data)	This paper
Top level	*Family Brand* e.g. Buick	*Parent anchoring brand* e.g Marlboro	*Brand family* e.g. JPS		*Brand* e.g. Marlboro
Level two	*Individual Brand* e.g. Buick Regal	*Brand family* e.g. Marlboro Fresh	*Brand/brand extension* e.g. JPS Duo	*House*[***] e.g. Marlboro Bright Leaf	*Brand family* e.g. Marlboro Bright Leaf
Level three	*Modifier* e.g. Buick Regal GS	*Brand variants* e.g. Marlboro Fresh brand variants: Marlboro Ice Blast or Marlboro Kretek Mint	*Products* e.g. JPS Duo Gold	*Brand* e.g. Marlboro Bright Leaf Platinum	*Brand variant* e.g Marlboro Bright Leaf Platinum
Bottom level	*Product Description* e.g. midsize luxury sport sedan automobile		*SKU* e.g. JPS Duo Gold 20s	*SKU* e.g. Marlboro Bright Leaf Platinum 10S multipack not price marked	*SKU* e.g. Marlboro Bright Leaf Platinum 10s multipack not price marked

[*] This reference[1] also discusses 'brand extensions' defined as where a brand is extended to a new type of product. In terms of tobacco this would be where an FM brand is extended to RYO e.g. Marlboro's RYO extension: Marlboro Gold Fine Cut Hand Rolling Tobacco.

[**]Obtained from an anonymous reviewer

[***] Nielsen only develops a new house when there is a brand variant at a different price point unlike other classifications. Thus PMI differentiate Marlboro into three brand families: Marlboro Red, Marlboro Gold and Marlboro Fresh[2]. Nielsen in the UK however do not differentiate Marlboro Red, Gold and Menthol. Applicable SKU are all listed as part of the Marlboro house/brand family. Only Marlboro Touch and Marlboro Bright Leaf, where SKU have lower prices, are listed as being in different houses/brand families.

Datasets

Segmentation analysis

Simplified datasets were used for the segmentation development (Table S2): We excluded price marked packs, wholesaler and retailer brands, RYO combipacks and MYO because prices per stick varied from mainstream SKUs and separated RYO and FM given their different pricing. For FM, Nielsen recommended only including data from SKUs distributed to >10% to retailers each month (Nielsen personal communication, 2016). This information was made available only from January 2013 onwards but a market share of 0.05% was found to be approximately equivalent for FM and for consistency we used the same market share criteria for RYO given that the distribution criteria was the same for FM and RYO. Thus we only included SKUs each month with >0.05% market share in each dataset.

Price and volume analysis

For further analyses the FM and RYO datasets were combined, all pack sizes and price marked packs were included and the restriction on market share was changed from 0.05% to >.008% (see footnote to Table S2 for more details). Two brands (one FM and one RYO), with idiosyncratic pricing, were excluded.

Table S2 SKUs included and excluded for segmentation and price analysis

	Segmentation development	Data analysis
Dataset		
RYO & FM cigarettes	Separate datasets	Merged to one dataset
Inclusions		
Market share	>0.05%[1]	>0.008%[2] for at least three months
FM pack sizes	17 to 20 stick packs[3]	All (except revenue analysis (Table 1 and Figure 5) where only 10 and 17 to 20 stick packs included)
RYO pack sizes	10g, 12.5g,25g,50g[4]	All (except revenue analysis (Table 1 and Figure 5) where only 10g, 12.5g,25g,50g included)
Exclusions		
Tobacco types	Combipacks[7], MYO[8]	Combipacks,[5, 6, 7] MYO[5, 6, 8]
Multipacks & single packs	Multipacks[7]	Multipacks[5, 6, 7]
Promotions	Pricemarked[7]	-
Companies	Retailer and wholesalers[7]	Retailer and wholesalers[5, 6, 7]
Brand families		Berkeley,[8] Swan[8]

[1] Chosen as approximately equivalent to distribution to 10% retailers. Nielsen use this distribution criteria as a guide to validity but it was only available to us from January 2013 so we used market share to be consistent throughout.
[2] Approximately equivalent to >.05% share in the RYO database so all SKU included in the segmentation analysis would be included in the price analysis.
[3] Only these packs included because prices per stick were similar
[4] Most common pack sizes and Amber Leaf was sold in these pack sizes
[5] Excluded due to low market share
[6] Excluded due to low distribution when this information became available
[7] Excluded due to lower or higher pricing than mainstream SKUs
[8] Excluded due to idiosyncratic pricing

Methodology for price segmentation development

We did not use statistical techniques for segmentation in case this led to oversimplification and obfuscated deliberate tobacco industry strategies. Tobacco industry pricing strategies are linked to brand marketing and thus consumer perceptions, and it is this, rather than statistical groupings, that influence consumer choices. Instead relative price positions of SKUs were analysed each month. This approach was used successfully in the PPACTE study.[3]

FM segmentation

For the first month of data, we allocated FM SKUs to segments based on the list of SKUs in each price segment developed in the PPACTE study[3] (premium, mid-price, economy and ultra low price) with the exception that the PPACTE economy segment was split into 'sub premium' and 'value' segments based on an apparent renaming of this segment names in the latest literature (Supplemental file Table S2). We listed all SKUs sold by price per stick for the first month together with their PPACTE segment. We then could calculate a price range for each segment. If there was no PPACTE segment we allocated the SKUs to the segment within which their price-point fell.

For subsequent months we again listed all SKUs sold, by price per stick, together with their segment from the previous month. New brand families were added to the segment within which their price fell. Problem SKUs, which appeared to cross segment boundaries, were flagged and addressed separately (see below)

Preliminary segmentation

From the literature and Nielsen data seven segments were originally identified: premium, vestigial mid-price (the brands identified as belonging to this segment in PPACTE either disappeared or were repriced during the data series), sub premium, value, original ultra low price (brand families in this segment at or near to the beginning of the data series), new ultra low price (brand families that appeared from 2011 onwards that were priced generally within the original ultra low price bracket) and sub ultra low price (brand families that appeared from 2012 onwards and were consistently priced below the ultra low price brand families).

Revised segmentation – flagged SKUs

All flagged SKUs were analysed in detail by two researchers to determine why they were not in the expected segment and rules were developed for allocating such SKUs where possible.

Reasons for SKUs not being in the expected segment (based both on data observations and the literature review) included: (a) delayed price increases (e.g. Dunhill); (b) high priced SKUs within one segment overlapping with low priced brand families within another segment (e.g. JPS Black, Sovereign Black, and Vogue Perle were priced between main sub premium brand families and value brand families for long patches); (c) low introductory pricing e.g. Marlboro Bright Leaf Platinum; (d) very low exit pricing– particularly in 2014 where the number of sticks per pack was changing from 20s to 19s (e.g. Richmond, Mayfair) and the old 20 packs were sold off very cheaply, (e) repricing a brand family into a different segment (eg. Superkings moved from vestigial midprice to premium or Berkeley where the segment declined several times); and (f) gimmicks e.g. click on or menthol which led to often to higher priced SKUs.

We produced graphs of brand families and segments in order to understand the segmentation better. Our visual analysis of flagged brands showed that although some SKUs might temporarily be priced within a neighbouring segment this generally occurred only for short periods and in the margins of the unexpected segment. As such it was simple to allocate each SKU to the segment

where it spent most time. There were two exceptions: first there was considerable overlap in price between the original ultra low price and new ultra low segments (these were therefore merged – see below); second it was impossible to allocate a clear segment to Berkeley brand families due to several marked price drops (this was therefore excluded – see below).

RYO

Initial graphical analysis of the RYO SKUs revealed that they were priced consistently above, in line with, or below one of the main traditional RYO brands- Amber Leaf. RYO SKUs were therefore listed by price per gram together with their segment based on their pricing relative to Amber Leaf. If SKUs appeared to move segments flags were used in the same way as for the FM SKUs.

Generalisation

The segmentation process revealed that Nielsen only differentiated FM brand families when they were introduced at different price points e.g. Marlboro Red and Marlboro Gold were not differentiated at brand family level whereas Marlboro Bright Leaf, which had lower prices, was differentiated. For RYO, Nielsen did not always differentiate brand families in this way. For example Amber Leaf Signature Blend SKUs were in the same brand family as the other Amber Leaf SKUs despite being more expensive. Thus FM SKUs were allocated to segments at the brand family level whereas RYO SKUs were allocated at the brand variant level. This difference may flow from there being fewer RYO SKUs: for example in 2015 there were 934 FM SKUs and 226 RYO SKUs.

The segment allocation of each FM brand family or RYO brand variant was extended to all other relevant pack sizes and multipack sizes and price marked SKU.

Final segments

For simplicity and for consistency between FM and RYO, the vestigial mid-price, sub premium and value segments were merged into a mid-price segment; the original and new ultra low segments were merged into a value segment and the sub ultra low price became the sub value segment. Thus for analysis the segments were FM premium, FM mid-price, FM value and FM sub value; RYO premium, RYO mid-price and RYO value.

Final Segment names (Table S3) were derived from the PPACTE literature review, the updated literature review, and particularly tobacco company segment names described in a key article in 2014[4].

The FM brand families and RYO brand variants were allocated as follows:

FM premium brand families were: B&H GOLD, CAMEL, DUNHILL, EMBASSY, MARLBORO, REGAL, ROTHMANS, SILK CUT, SOLO, SUPERKINGS, CONSULATE, LUCKY STRIKE, MORE, PARK DRIVE, PETER STUYVESANT, PLAYERS NAVY CUT, SENIOR SERVICE, ST MORITZ, VOGUE, WOODBINE.

The following brand families were in the FM mid-price segment and they are subdivided into their preliminary segments: vestigial mid-price: CLUB, RAFFLES, NATURAL AMERICAN SPIRIT; sub premium, B&H SILVER, LAMBERT & BUTLER, B&H DUAL, B&H BLACK, B&H WHITE, CRAVEN A, VOGUE PERLE; value: DORCHESTER, JPS BLACK, JPS WHITE, MAYFAIR, RICHMOND, ROYALS, SOVEREIGN, MARLBORO BRIGHT LEAF.

The following brand families were in the FM value segment and they are subdivided into their original segments: original ultra low price: JPS, PALL MALL, RONSON, SPECIALS, STERLING, WINDSOR, WINSTON, MAXIM; new ultra low price: CHESTERFIELD, SOVEREIGN BLUE, B&H BLUE, LAMBERT & BUTLER BLUE, MARLBORO TOUCH.

The FM sub value brand families were: CARLTON, ROTHMANS OF LONDON, PLAYERS, ROTHMANS BLEND 55, ALLURE

The following brand families were excluded: BERKELEY KING SIZE and BERKELEY SUPERKINGS were excluded due to idiosyncratic pricing and other brand families were excluded due to being retailer and wholesaler brands as they had <10% distribution to retailers where this information was available (BALMORAL, BEAUMONT, GOLDMARK, KINGSMEN, LONDIS, NO 3, PARK ROAD, RED BAND, SELECT, SILVER STRAND, SKY).

RYO premium brand variants were GOLDEN VIRGINIA, AMBER LEAF SIGNATURE BLEND, BENSON & HEDGES GOLD, DRUM, DRUM ADDITIVE FREE, DRUM GOLD, NATURAL AMERICAN SPIRIT, OLD HOLBORN.

RYO mid-price brand variants were AMBER LEAF, CUTTERS CHOICE, CUTTERS CHOICE A TRUE BLEND, AMBER LEAF BLONDE, BENSON & HEDGES SILVER, CUTTERS CHOICE EXTRA SMOOTH, CUTTERS CHOICE GOLD, GOLDEN VIRGINIA SMOOTH, MARLBORO GOLD, SAMSON, OLD HOLBORN YELLOW, CUTTERS CHOICE EXQUISITE BLEND, SAMSON VIRGINIA

RYO value brand variants were GOLD LEAF, HOLBORN SMOOTH TASTE, JOHN PLAYER SPECIAL SILVER, STERLING, PALL MALL, CARLTON, ROL, SALSA VIRGINIA BLEND, THE TURNER, URBAN, ASHFORD GOLD BRIGHT VIRGINIA, MANITOU GOLD, PUEBLO, PLAYERS RED VOLUME TOBACCO

The following were excluded: Make Your Own (MYO) (CONCEPT TOBACCO, JOHN PLAYER SPECIAL MAKE YOUR OWN), combi packs: (AMBER LEAF 3 IN 1, GOLDEN VIRGINIA HANDY PACK, GOLDEN VIRGINIA SMOOTH HANDY PACK); retailer & wholesaler (PRIVATE LABEL, RED BAND) and an accessories brand SWAN which had idiosyncratic pricing.

Table S3 Segmentation

Previous segmentation (PPACTE Study)	Preliminary segmentation		Final segmentation
2001-10 segments[1]	2009-15 segments[2]		2009-15 segments used in the analysis[2]
FM premium	FM premium<br>e.g. Embassy	Premium	FM premium
FM mid-price	FM vestigial mid-price[3]<br>e.g. Club	-	FM mid-price
FM economy	FM sub premium<br>e.g. Lambert & Butler	Sub premium, mid-price, premium	
	FM value<br>e.g. Mayfair	Value, mid-price economy, discount	
FM ultra low price	FM ultra low price – original[4]<br>e.g. Pall Mall	Economy, value, budget, ultra discount, ultra low price	FM value
	FM ultra low price- new[5]<br>e.g. Sovereign Blue	Value, sub £6	
	FM sub ultra low price[6]<br>e.g. Players	Economy, sub economy, sub value	FM sub value
RYO	RYO premium<br>e.g. Golden Virginia	Premium	RYO premium
	RYO mid-price<br>e.g. Amber Leaf	Mid-price, mid market, sub premium	RYO mid-price
	RYO value<br>e.g. Gold Leaf	Value, economy, lower cost	RYO value

[1]These segments were identified by the PPACTE project [3]

[2]Segmentation identified through the commercial literature review and Nielsen data

[3]This segment was named 'vestigial' mid-price because most of the PPACTE mid-price brands were discontinued or repositioned to other segments

[4]Original ultra low price brand families were in existence in in the first month of data

[5]New ultra low price brand families appeared from 2011 onwards. They were priced similarly to original ultra low price brand families and mostly towards the lower range of original ultra low priced brands

[6] Sub ultra low price brands appeared from 2012 onwards. They were generally the lowest priced brands and priced below original ultra low priced brands

Supplemental file 2 Calculation of net revenue

The net revenue for each pack was estimated by calculating the tobacco taxes (specific (plus ad valorem for FM only)) and VAT applicable to each pack and deducting this from the nominal pack price. VAT was calculated as the applicable percentage of the price of the pack applicable each month[5]. Specific taxes were calculated as the applicable rate for each month multiplied by the pack size (in grams or sticks) divided per 1000 (as the specific rate is per 1000 sticks or 1000 kilograms) [6]

Ad valorem calculation is more complex. The HMRC calculate only one ad valorem rate for each brand [41] (price marked packs do not incur a separate tax rate [7]) and the rate is calculated using the recommended retail price (RRP) of 20 stick packs and then applied proportionately to different pack sizes [8]. There were three issues that arose for the calculation of ad valorem tax from HMRC's reliance on 20 packs: firstly Nielsen data revealed that some 20 packs were being sold off very cheaply (they were cheaper than 19 stick packs) from 2014 onwards. This implied that the sales price was substantially below the RRP. Second, some brands (particularly from 2014 onwards) did not have a 20 stick variant (43 of the 352 FM brands recorded in the Nielsen data never had a recorded 20 stick variant (12%) and others lost their 20 stick variant). Third, in some cases there might be an existing 20 stick variant which had been excluded due to insufficient market share for data to be reliable.

We therefore estimated ad valorem tax in the following way. First, we restricted the analysis to the most common pack sizes (10, 17, 18, 19 and 20 sticks) so 20 sticks were the largest pack size and theoretically should thus be the most expensive if they were being sold at the RRP. Second, we identified which was the most expensive brand variant each month for each brand and its pack size. If it was the 20 pack we used this price to calculate ad valorem. If it was not the 20 pack but there was a 20 stick variant available at some point in the data series then we imputed the pack price of an imaginary 20 stick variant using the price of the highest priced available variant and used the imputed price to calculate the ad valorem tax. The brand's ad valorem was calculated by the pack price multiplied by the applicable monthly ad valorem rate. For packs containing less than 20 sticks, the ad valorem tax was calculated pro rata to the 20 stick equivalent.

Supplemental file 3 Segment analysis

The tables below provide data supporting the figures in the main article. In Tables A4, A5,A8 and A10 price points are shown in January each year and the final data point (December 2015). Note that December 2015 data, particularly for volumes, should not be directly compared with other months due to seasonal cyclical variations: higher volumes sold compared with January 2015 may be the result of higher sales during the Christmas holiday period compared with early in the new year.

Recall the criteria for inclusion: SKUs needed to reach market share >.008% for three months. Price marked packs were included in the segmentation analysis. The following were excluded: SKU sold in multipacks, retailer and wholesaler brands, MYO and combipacks and two idiosyncratically priced brands (Berkeley and Swan) due to different pricing strategies from main stream SKU and limited distribution to retailers.

Table S4 Real weighted average price and volume changes over time (packs and sticks) by segment

	Jan-09	Jan-10	Jan-11	Jan-12	Jan-13	Jan-14	Jan-15	Dec-15
Price per stick								
FM premium	£0.29	£0.30	£0.31	£0.33	£0.35	£0.37	£0.40	£0.42
FM mid-price	£0.25	£0.26	£0.27	£0.29	£0.31	£0.33	£0.35	£0.36
FM value	£0.22	£0.22	£0.23	£0.26	£0.28	£0.30	£0.31	£0.33
FM sub value					£0.26	£0.27	£0.28	£0.29
RYO premium	£0.12	£0.12	£0.12	£0.14	£0.14	£0.15	£0.16	£0.17
RYO mid-price	£0.11	£0.11	£0.11	£0.13	£0.13	£0.14	£0.15	£0.15
RYO value	£0.10	£0.10	£0.11	£0.12	£0.12	£0.13	£0.13	£0.14
Difference in stick price FM premium to RYO value	£0.15	£0.16	£0.16	£0.17	£0.19	£0.20	£0.22	£0.22
Price per pack								
FM premium	£4.78	£4.97	£5.15	£5.32	£5.67	£5.99	£6.50	£6.76
FM mid-price	£4.05	£4.19	£4.33	£4.60	£4.87	£5.11	£5.36	£5.61
FM value	£3.80	£3.81	£3.97	£4.34	£4.65	£4.79	£5.05	£5.10
FM sub value					£4.75	£4.66	£4.71	£4.68
RYO premium	£4.42	£4.75	£4.96	£5.42	£5.81	£6.16	£6.63	£6.97
RYO mid-price	£3.64	£3.96	£4.13	£4.67	£4.96	£5.24	£5.51	£5.81
RYO value	£3.04	£2.92	£3.45	£3.94	£4.26	£4.16	£4.24	£4.28
Difference in pack price FM premium to RYO value	£1.74	£2.05	£1.70	£1.38	£1.41	£1.83	£2.26	£2.48

Table S5 Annual volumes of sticks and packs (millions)

	2009	2010	2011	2012	2013	2014	2015	% change 2009-2015
Sticks								
All[1] Sticks								
FM	41,831	40,951	39,148	36,545	34,055	32,883	30,710	-17
RYO	8,671	9,691	10,830	11,262	11,824	11,980	11,775	46
MYO	20	17	24	78	114	119	108	437
total	50,522	50,659	50,003	47,885	45,993	44,982	42,593	-13
Analysed by price segment[2]								
FM	34,886	34,276	33,274	31,353	29,890	29,477	27,871	-15
RYO	8,671	9,691	10,804	11,143	11,580	11,523	11,158	29
total	43,557	43,967	44,078	42,497	41,469	41,000	39,029	-9
% analysed								
FM	83%	84%	85%	86%	88%	90%	91%	
RYO	100%	100%	100%	99%	98%	96%	95%	
total	86%	87%	88%	89%	90%	91%	92%	
Packs								
All[1] packs	2009	2010	2011	2012	2013	2014	2015	
FM	2,277	2,233	2,179	2,063	1,954	1,932	1,828	-15
RYO	239	262	291	303	323	336	335	40
MYO	1	1	1	2	2	2	2	132
total	2,517	2,495	2,470	2,367	2,279	2,270	2,165	-12
Analysed by price segment[2]								
FM	2,143	2,104	2,061	1,951	1,869	1,860	1,767	-14
RYO	239	262	290	298	312	314	305	28
total	2,382	2,366	2,350	2,250	2,181	2,174	2,073	-11

[1] Includes all SKU >.008% market share

[2] Includes all SKU >.008% market share (so long as this market share was achieved for three months) but excludes multipacks, retailer & wholesaler, MYO & combipacks, Berkeley & Swan

Table S6 Annual volumes of sticks and packs (millions) by price segment

	2009	2010	2011	2012	2013	2014	2015	% change 2009-15	% change 2012-15
Sticks									
FM premium	9,904	8,739	7,587	6,737	5,927	5,340	4,509	-54	-33
FM mid-price	20,022	18,207	15,962	13,702	11,758	9,783	7,822	-61	-43
FM value	4,960	7,329	9,725	10,630	10,987	11,577	11,217	126	6
FM sub value				284	1,217	2,777	4,323		1422
RYO premium	4,964	4,793	4,407	3,889	3,431	3,154	2,841	-43	-27
RYO mid-price	3,374	4,419	5,512	5,891	6,278	6,182	6,012	78	2
RYO value	333	479	885	1,363	1,871	2,187	2,305	592	69
Packs									
FM premium	603	532	466	415	367	332	279	-54	-33
FM mid-price	1,251	1,143	1,013	877	758	641	514	-59	-41
FM value	288	430	582	643	673	722	708	146	10
FM sub value				16	70	165	266		1586
RYO premium	130	122	111	98	85	77	69	-47	-30
RYO mid-price	97	124	152	161	171	168	163	68	1
RYO value	12	15	27	40	56	68	74	519	86

Table S7 Annual volumes of packs (millions) by price segment and pack size and market share

	Annual pack sales (millions)							Market share						
	2009	2010	2011	2012	2013	2014	2015	2009	2010	2011	2012	2013	2014	2015
FM premium 10 stick	215	187	171	154	140	130	108	36%	35%	37%	38%	38%	39%	39%
FM premium 20 stick	386	341	291	254	225	201	171	64%	65%	63%	62%	62%	61%	61%
FM mid-price 10 stick	504	468	428	376	338	290	227	41%	42%	43%	45%	46%	46%	45%
FM mid-price 19 stick						112	166	0%	0%	0%	0%	0%	18%	33%
FM mid-price 20 stick	728	658	565	467	404	227	112	59%	58%	57%	55%	54%	36%	22%
FM value 10 stick	79	122	185	220	236	253	231	27%	28%	32%	34%	35%	35%	33%
FM value 17 stick							18	0%	0%	0%	0%	0%	0%	3%
FM value 18 stick	1					5	135	0%	0%	0%	0%	0%	1%	19%
FM value 19 stick	12	43	60	75	107	324	316	4%	10%	10%	11%	16%	45%	45%
FM value 20 stick	197	264	336	357	329	140	8	68%	61%	58%	55%	49%	19%	1%
FM sub value 10 stick				3	17	39	67				20%	24%	23%	25%
FM sub value 18 stick						14	122				0%	0%	8%	46%
FM sub value 19 stick					18	106	77				0%	26%	64%	29%
FM sub value 20 stick				13	35	7	<1				80%	49%	4%	0%
RYO premium 10g						<1	1	0%	0%	0%	0%	0%	1%	1%
RYO premium 12.5g	79	73	67	58	51	44	37	62%	60%	60%	60%	59%	57%	54%
RYO premium 25g	39	37	33	29	26	24	23	30%	31%	30%	30%	30%	32%	34%
RYO premium 50g	10	11	10	9	9	8	7	8%	9%	9%	10%	10%	11%	11%
RYO mid-price 10g	1	2	2	4	6	13	25	1%	2%	2%	2%	4%	8%	17%
RYO mid-price 12.5g	70	86	102	107	110	98	76	72%	69%	68%	66%	64%	60%	51%
RYO mid-price 25g	20	28	36	39	42	40	37	21%	23%	24%	24%	25%	25%	25%
RYO mid-price 50g	6	8	11	13	13	12	12	6%	7%	7%	8%	8%	7%	8%
RYO value 10g					5	13	9	0%	0%	0%	0%	9%	24%	21%
RYO value 12.5g	<1	8	20	28	32	28	17	82%	77%	75%	71%	61%	50%	42%
RYO value 25g	<1	2	6	10	13	12	11	2%	21%	22%	24%	24%	22%	28%
RYO value 50g	<1	<1	1	2	3	3	4	16%	3%	3%	5%	5%	5%	9%

Table S8 Real weighted average price per pack by price segment and pack size

	Jan-09	Jan-10	Jan-11	Jan-12	Jan-13	Jan-14	Jan-15	Dec-15
FM premium 10 stick	£3.02	£3.12	£3.25	£3.41	£3.63	£3.86	£4.23	£4.39
FM premium 17-20 stick	£5.75	£5.95	£6.18	£6.47	£6.89	£7.33	£7.93	£8.18
FM mid-price 10 stick	£2.61	£2.69	£2.81	£3.03	£3.25	£3.43	£3.69	£3.85
FM mid-price 17-20 stick	£4.97	£5.14	£5.37	£5.76	£6.16	£6.49	£6.69	£6.91
FM value 10 stick	£2.28	£2.25	£2.39	£2.68	£2.93	£3.08	£3.31	£3.44
FM value 17-20 stick	£4.32	£4.40	£4.62	£5.13	£5.52	£5.77	£5.89	£5.87
FM sub value 10 stick					£2.71	£2.80	£2.87	£2.89
FM sub value 17-20 stick					£5.24	£5.23	£5.26	£5.33
RYO premium 10g							£3.21	£3.33
RYO premium 12.5g	£2.95	£3.06	£3.17	£3.47	£3.68	£3.87	£4.15	£4.31
RYO premium 25g	£5.80	£5.96	£6.15	£6.73	£7.13	£7.49	£7.91	£8.16
RYO premium 50g	£11.29	£11.22	£12.02	£13.13	£14.00	£14.69	£15.52	£16.00
RYO mid-price 10g	£2.23	£2.37	£2.30	£2.60	£2.71	£2.91	£3.08	£3.26
RYO mid-price 12.5g	£2.70	£2.79	£2.89	£3.24	£3.44	£3.61	£3.84	£3.98
RYO mid-price 25g	£5.25	£5.40	£5.60	£6.25	£6.66	£6.99	£7.38	£7.60
RYO mid-price 50g	£10.21	£10.54	£10.97	£12.21	£13.04	£13.71	£14.51	£14.96
RYO value 10g						£2.57	£2.70	£2.77
RYO value 12.5g	£2.69	£2.56	£2.70	£2.99	£3.14	£3.24	£3.46	£3.54
RYO value 25g			£5.21	£5.79	£6.05	£6.24	£6.58	£6.69
RYO value 50g		£7.49	£9.43	£10.55	£11.63	£12.18	£12.83	£13.02

Table S9a Annual total volume of sticks and packs sold (millions) by price segment and price marked packaging (pmp)

	2012			2013			2014			2015		
	non pricemarked	pmp	% pmp	non pricemarked	pmp	% pmp	non pricemarked	pmp	% pmp	non pricemarked	pmp	% pmp
Sticks												
FM premium	6,598	139	2.1%	5,907	21	0.4%	5,340	-	0%	4,509	-	0%
FM mid-price	9,307	4396	32.1%	7,572	4186	35.6%	6,147	3637	37.2%	4,956	2866	36.6%
FM value	5,509	5121	48.2%	5,003	5984	54.5%	4,530	7047	60.9%	4,644	6573	58.6%
FM sub value	91	193	68.0%	382	835	68.6%	908	1868	67.3%	1,571	2752	63.7%
RYO premium	2,833	1056	27.2%	2,659	772	22.5%	2,437	716	22.7%	2,186	655	23.1%
RYO mid-price	4,274	1617	27.4%	4,007	2271	36.2%	3,654	2529	40.9%	3,315	2697	44.9%
RYO value	731	632	46.4%	948	923	49.3%	1,073	1113	50.9%	1,158	1147	49.8%
Packs												
FM premium	409	7	1.7%	366	1	0.3%	332	-	0%	279	-	0%
FM mid-price	600	277	31.6%	489	270	35.6%	401	240	37.4%	323	190	37.0%
FM value	331	312	48.5%	305	367	54.6%	289	433	60.0%	297	411	58.1%
FM sub value	5	11	68.8%	20	50	71.4%	51	113	68.9%	93	173	65.0%
RYO premium	65	33	33.7%	64	21	24.7%	59	19	24.4%	52	17	24.6%
RYO mid-price	112	49	30.4%	103	68	39.8%	95	73	43.5%	87	76	46.6%
RYO value	20	19	48.7%	26	30	53.6%	30	38	55.9%	33	41	55.4%

Table S9b Annual total volume of sticks and packs sold (millions) by pack size and price marked packaging (pmp)

	2012			2013			2014			2015		
	non pricemarked	pmp	% pmp	non pricemarked	pmp	% pmp	non pricemarked	pmp	% pmp	non pricemarked	pmp	% pmp
Sticks												
FM 10 sticks	5,316	2249	29.7%	4,690	2637	36.0%	4,313	2804	39.4%	3,692	2640	41.7%
FM 17 to 20 sticks	15,932	7469	31.9%	13,993	8257	37.1%	12,473	9643	43.6%	11,891	9476	44.3%
RYO 10g	28	49	63.6%	55	167	75.2%	195	342	63.7%	323	362	52.8%
RYO 12.5g	2,966	1851	38.4%	2,882	1928	40.1%	2,484	1773	41.6%	1,868	1405	42.9%
RYO 25g	2,811	1047	27.1%	2,712	1307	32.5%	2,487	1363	35.4%	2,169	1378	38.8%
RYO 50g	2,000	380	16.0%	1,946	487	20.0%	1,703	615	26.5%	1,575	687	30.4%
Packs												
FM 10 sticks	531	225	29.8%	469	264	36.0%	431	280	39.4%	369	264	41.7%
FM 17 to 20 sticks	798	375	32.0%	702	417	37.3%	635	500	44.1%	619	506	45.0%
RYO 10g	1	2	66.7%	3	8	72.7%	10	17	63.0%	16	18	52.9%
RYO 12.5g	119	74	38.3%	115	77	40.1%	99	71	41.8%	75	56	42.7%
RYO 25g	56	21	27.3%	54	26	32.5%	50	27	35.1%	43	28	39.4%
RYO 50g	20	4	16.7%	19	5	20.8%	17	6	26.1%	16	7	30.4%

Table S10 Real weighted average price per pack by pricemarking (PMP)

	January 2012			January 2013			January 2014			January 2015			December 2015		
	non price-marked	pmp	% difference	non price-marked	pmp	% difference	non price-marked	pmp	% differenc e	non price-marked	pmp	% difference	non price-marked	pmp	% differenc e
FM premium	£5.30	£6.27	-15.5	£5.66	£6.85	-17.4	£5.99			£6.50			£6.76		
FM mid-price	£4.60	£4.60	0	£4.93	£4.78	3.1	£5.19	£4.96	4.6	£5.47	£5.17	5.8	£5.76	£5.34	7.9
FM value	£4.42	£4.25	4.0	£4.75	£4.56	4.2	£4.87	£4.73	3.0	£5.07	£5.03	0.8	£5.21	£5.01	4.0
FM sub value				£5.19	£4.54	14.3	£4.92	£4.54	8.4	£5.00	£4.57	9.4	£4.98	£4.51	10.4
RYO premium	£6.02	£4.35	38.4	£6.05	£5.07	19.3	£6.30	£5.69	10.7	£6.81	£6.09	11.8	£7.16	£6.37	12.4
RYO mid-price	£4.89	£4.13	18.4	£5.29	£4.41	20.0	£5.54	£4.76	16.4	£5.74	£5.24	9.5	£6.08	£5.52	10.1
RYO value	£4.33	£3.59	20.6	£4.68	£3.89	20.3	£4.63	£3.79	22.2	£4.82	£3.79	27.2	£4.86	£3.83	26.9

Table S11 Change in net revenue per pack from budget month by price segment (typical pack size)

	Jan	Feb	Mar	April	May	June	July	Aug	Sept	Oct	Nov	Dec
2009												
FM premium*	£1.24	£1.26	£1.28	£1.26	£1.27	£1.26	£1.27	£1.26	£1.26	£1.26	£1.26	£1.27
FM economy	£0.77	£0.78	£0.79	£0.79	£0.78	£0.79	£0.80	£0.80	£0.80	£0.80	£0.81	£0.82
FM value					£0.33	£0.37	£0.40	£0.39	£0.40	£0.41	£0.41	£0.37
FM sub value												
RYO premium	£1.03	£0.98	£0.98	£0.98	£0.97	£0.97	£0.97	£0.98	£0.98	£0.98	£0.98	£0.99
RYO mid-price	£0.82	£0.76	£0.79	£0.79	£0.77	£0.78	£0.80	£0.80	£0.79	£0.79	£0.80	£0.81
RYO value	£0.80	£0.58	£0.71	£0.71	£0.60	£0.60	£0.52	£0.60	£0.55	£0.55	£0.54	£0.54
2010												
FM premium	£1.25	£1.26	£1.26	£1.23	£1.24	£1.24	£1.25	£1.25	£1.25	£1.28	£1.29	£1.30
FM economy	£0.78	£0.79	£0.81	£0.77	£0.78	£0.80	£0.80	£0.81	£0.81	£0.82	£0.84	£0.84
FM value	£0.32	£0.34	£0.35	£0.31	£0.33	£0.35	£0.36	£0.37	£0.37	£0.38	£0.38	£0.38
FM sub value												
RYO premium	£1.00	£1.01	£1.01	£1.00	£1.01	£1.01	£1.02	£1.01	£1.02	£1.05	£1.05	£1.05
RYO mid-price	£0.78	£0.80	£0.81	£0.78	£0.79	£0.81	£0.82	£0.82	£0.82	£0.82	£0.83	£0.83
RYO value	£0.59	£0.59	£0.66	£0.58	£0.60	£0.62	£0.63	£0.66	£0.66	£0.67	£0.68	£0.68
2011												
FM premium	£1.22	£1.23	£1.24	£1.19	£1.22	£1.23	£1.23	£1.23	£1.24	£1.28	£1.29	£1.29
FM economy	£0.77	£0.78	£0.80	£0.68	£0.70	£0.73	£0.76	£0.76	£0.77	£0.79	£0.81	£0.82
FM value	£0.31	£0.34	£0.35	£0.17	£0.21	£0.28	£0.35	£0.35	£0.33	£0.32	£0.35	£0.40
FM sub value												
RYO premium	£1.01	£1.01	£1.02	£0.92	£0.96	£0.96	£0.98	£0.99	£1.00	£1.02	£1.04	£1.04
RYO mid-price	£0.79	£0.81	£0.83	£0.71	£0.73	£0.75	£0.79	£0.81	£0.83	£0.83	£0.83	£0.85
RYO value	£0.64	£0.65	£0.67	£0.49	£0.49	£0.48	£0.51	£0.53	£0.59	£0.64	£0.66	£0.67
2012												
FM premium	£1.30	£1.29	£1.34	£1.26	£1.28	£1.29	£1.33	£1.34	£1.38	£1.41	£1.41	£1.41
FM economy	£0.84	£0.84	£0.87	£0.73	£0.74	£0.77	£0.83	£0.84	£0.88	£0.89	£0.91	£0.93
FM value	£0.40	£0.41	£0.42	£0.26	£0.28	£0.30	£0.30	£0.34	£0.41	£0.43	£0.44	£0.45
FM sub value												
RYO premium	£1.05	£1.05	£1.08	£1.02	£1.03	£1.04	£1.08	£1.09	£1.10	£1.10	£1.10	£1.11
RYO mid-price	£0.87	£0.87	£0.89	£0.82	£0.82	£0.84	£0.86	£0.87	£0.89	£0.90	£0.90	£0.91
RYO value	£0.67	£0.67	£0.68	£0.58	£0.59	£0.61	£0.64	£0.65	£0.67	£0.67	£0.67	£0.69
2013												
FM premium	£1.42	£1.41	£1.43	£1.40	£1.43	£1.44	£1.46	£1.47	£1.50	£1.55	£1.58	£1.58
FM economy	£0.95	£0.95	£0.96	£0.86	£0.87	£0.89	£0.94	£0.94	£0.96	£0.99	£1.01	£1.03
FM value	£0.45	£0.46	£0.47	£0.35	£0.37	£0.40	£0.44	£0.41	£0.41	£0.42	£0.46	£0.51
FM sub value	£0.44	£0.47	£0.46	£0.33	£0.31	£0.30	£0.37	£0.36	£0.35	£0.36	£0.37	£0.38
RYO premium	£1.13	£1.13	£1.13	£1.09	£1.10	£1.11	£1.14	£1.15	£1.15	£1.19	£1.21	£1.21
RYO mid-price	£0.94	£0.94	£0.95	£0.90	£0.90	£0.92	£0.94	£0.94	£0.95	£0.97	£0.98	£1.01
RYO value	£0.70	£0.68	£0.68	£0.55	£0.58	£0.60	£0.61	£0.63	£0.65	£0.66	£0.68	£0.71
2014												
FM premium	£1.60	£1.62	£1.66	£1.61	£1.65	£1.68	£1.72	£1.74	£1.78	£1.80	£1.83	£1.84
FM economy	£1.06	£1.06	£1.08	£0.98	£1.00	£1.05	£1.08	£1.10	£1.14	£1.17	£1.19	£1.20
FM value	£0.52	£0.53	£0.57	£0.46	£0.48	£0.54	£0.58	£0.60	£0.62	£0.62	£0.64	£0.67
FM sub value	£0.38	£0.40	£0.43	£0.31	£0.31	£0.30	£0.30	£0.29	£0.31	£0.33	£0.35	£0.35
RYO premium	£1.22	£1.22	£1.25	£1.22	£1.22	£1.22	£1.24	£1.27	£1.31	£1.33	£1.34	£1.34
RYO mid-price	£1.02	£1.02	£1.03	£0.98	£0.98	£0.99	£1.00	£1.01	£1.04	£1.06	£1.06	£1.09
RYO value	£0.72	£0.73	£0.76	£0.72	£0.73	£0.73	£0.74	£0.74	£0.77	£0.79	£0.79	£0.81
2015												
FM premium	£1.85	£1.86	£1.87	£1.83	£1.86	£1.86	£1.87	£1.86	£1.90	£1.93	£1.95	£1.95
FM economy	£1.22	£1.23	£1.25	£1.19	£1.23	£1.25	£1.27	£1.28	£1.29	£1.31	£1.33	£1.33
FM value	£0.71	£0.72	£0.73	£0.65	£0.65	£0.65	£0.65	£0.65	£0.66	£0.66	£0.66	£0.66
FM sub value	£0.36	£0.34	£0.34	£0.26	£0.27	£0.28	£0.29	£0.29	£0.32	£0.35	£0.37	£0.38
RYO premium	£1.37	£1.38	£1.39	£1.36	£1.37	£1.37	£1.37	£1.38	£1.39	£1.43	£1.46	£1.47
RYO mid-price	£1.12	£1.13	£1.14	£1.10	£1.11	£1.12	£1.13	£1.13	£1.16	£1.18	£1.18	£1.20
RYO value	£0.82	£0.83	£0.86	£0.81	£0.80	£0.81	£0.82	£0.81	£0.82	£0.83	£0.84	£0.84

*FM premium and FM mid-price are 20 stick packs, FM value and FM sub value are 19 stick packs, RYO is 12.5g packs.

Table S12 Change in net revenue per pack from budget month by price segment (typical pack size)

Budget year	April	May	June	July	Aug	Sept	Oct	Nov	Dec	Jan	Feb	Mar
2009*												
FM premium**		£0.01	£0.00	£0.01	£0.00	£0.00	£0.00	£0.00	£0.01	-£0.01	£0.00	£0.00
FM economy		-£0.01	£0.00	£0.01	£0.01	£0.01	£0.01	£0.02	£0.03	-£0.01	£0.00	£0.02
FM value		-£0.02	-£0.01	£0.00	£0.00	£0.00	£0.01	£0.01	£0.02	-£0.02	-£0.01	£0.01
FM sub value												
RYO premium		-£0.01	-£0.01	-£0.01	£0.00	£0.00	£0.00	£0.00	£0.01	£0.02	£0.03	£0.03
RYO mid-price		-£0.02	-£0.01	£0.01	£0.01	£0.00	£0.00	£0.01	£0.02	-£0.01	£0.01	£0.02
RYO value		-£0.11	-£0.11	-£0.19	-£0.11	-£0.16	-£0.16	-£0.17	-£0.17	-£0.12	-£0.12	-£0.05
2010												
FM premium	-£0.03	-£0.02	-£0.02	-£0.01	-£0.01	-£0.01	£0.02	£0.03	£0.04	-£0.04	-£0.03	-£0.02
FM economy	-£0.04	-£0.03	-£0.01	-£0.01	£0.00	£0.00	£0.01	£0.03	£0.03	-£0.04	-£0.03	-£0.01
FM value	-£0.04	-£0.02	£0.00	£0.01	£0.02	£0.02	£0.03	£0.03	£0.03	-£0.04	-£0.01	£0.00
FM sub value												
RYO premium	-£0.01	£0.00	£0.00	£0.01	£0.00	£0.01	£0.04	£0.04	£0.04	£0.00	£0.00	£0.01
RYO mid-price	-£0.03	-£0.02	£0.00	£0.01	£0.01	£0.01	£0.01	£0.02	£0.02	-£0.02	£0.00	£0.02
RYO value	-£0.08	-£0.06	-£0.04	-£0.03	£0.00	£0.00	£0.01	£0.02	£0.02	-£0.02	-£0.01	£0.01
2011												
FM premium	-£0.05	-£0.02	-£0.01	-£0.01	-£0.01	£0.00	£0.04	£0.05	£0.05	£0.06	£0.05	£0.10
FM economy	-£0.12	-£0.10	-£0.07	-£0.04	-£0.04	-£0.03	-£0.01	£0.01	£0.02	£0.04	£0.04	£0.07
FM value	-£0.18	-£0.14	-£0.07	£0.00	£0.00	-£0.02	-£0.03	£0.00	£0.05	£0.05	£0.06	£0.07
FM sub value												
RYO premium	-£0.10	-£0.06	-£0.06	-£0.04	-£0.03	-£0.02	£0.00	£0.02	£0.02	£0.03	£0.03	£0.06
RYO mid-price	-£0.12	-£0.10	-£0.08	-£0.04	-£0.02	£0.00	£0.00	£0.00	£0.02	£0.04	£0.04	£0.06
RYO value	-£0.18	-£0.18	-£0.19	-£0.16	-£0.14	-£0.08	-£0.03	-£0.01	£0.00	£0.00	£0.00	£0.01
2012												
FM premium	-£0.08	-£0.06	-£0.05	-£0.01	£0.00	£0.04	£0.07	£0.07	£0.07	£0.08	£0.07	£0.09
FM economy	-£0.14	-£0.13	-£0.10	-£0.04	-£0.03	£0.01	£0.02	£0.04	£0.06	£0.08	£0.08	£0.09
FM value	-£0.16	-£0.14	-£0.12	-£0.12	-£0.08	-£0.01	£0.01	£0.02	£0.03	£0.03	£0.04	£0.05
FM sub value												
RYO premium	-£0.06	-£0.05	-£0.04	£0.00	£0.01	£0.02	£0.02	£0.02	£0.03	£0.05	£0.05	£0.05
RYO mid-price	-£0.07	-£0.07	-£0.05	-£0.03	-£0.02	£0.00	£0.01	£0.01	£0.02	£0.05	£0.05	£0.06
RYO value	-£0.10	-£0.09	-£0.07	-£0.04	-£0.03	-£0.01	-£0.01	-£0.01	£0.01	£0.02	£0.00	£0.00
2013												
FM premium	-£0.03	£0.00	£0.01	£0.03	£0.04	£0.07	£0.12	£0.15	£0.15	£0.17	£0.19	£0.23
FM economy	-£0.10	-£0.09	-£0.07	-£0.02	-£0.02	£0.00	£0.03	£0.05	£0.07	£0.10	£0.10	£0.12
FM value	-£0.12	-£0.10	-£0.07	-£0.03	-£0.06	-£0.06	-£0.05	-£0.01	£0.04	£0.05	£0.06	£0.10
FM sub value	-£0.13	-£0.15	-£0.16	-£0.09	-£0.10	-£0.11	-£0.10	-£0.09	-£0.08	-£0.08	-£0.06	-£0.03
RYO premium	-£0.04	-£0.03	-£0.02	£0.01	£0.02	£0.02	£0.06	£0.08	£0.08	£0.09	£0.09	£0.12
RYO mid-price	-£0.05	-£0.05	-£0.03	-£0.01	-£0.01	£0.00	£0.02	£0.03	£0.06	£0.07	£0.07	£0.08
RYO value	-£0.13	-£0.10	-£0.08	-£0.07	-£0.05	-£0.03	-£0.02	£0.00	£0.03	£0.04	£0.05	£0.08
2014												
FM premium	-£0.05	-£0.01	£0.02	£0.06	£0.08	£0.12	£0.14	£0.17	£0.18	£0.19	£0.20	£0.21
FM economy	-£0.10	-£0.08	-£0.03	£0.00	£0.02	£0.06	£0.09	£0.11	£0.12	£0.14	£0.15	£0.17
FM value	-£0.11	-£0.09	-£0.03	£0.01	£0.03	£0.05	£0.05	£0.07	£0.10	£0.14	£0.15	£0.16
FM sub value	-£0.12	-£0.12	-£0.13	-£0.13	-£0.14	-£0.12	-£0.10	-£0.08	-£0.08	-£0.07	-£0.09	-£0.09
RYO premium	-£0.03	-£0.03	-£0.03	-£0.01	£0.02	£0.06	£0.08	£0.09	£0.09	£0.12	£0.13	£0.14
RYO mid-price	-£0.05	-£0.05	-£0.04	-£0.03	-£0.02	£0.01	£0.03	£0.03	£0.06	£0.09	£0.10	£0.11
RYO value	-£0.04	-£0.03	-£0.03	-£0.02	-£0.02	£0.01	£0.03	£0.03	£0.05	£0.06	£0.07	£0.10
2015												
FM premium	-£0.04	-£0.01	-£0.01	£0.00	-£0.01	£0.03	£0.06	£0.08	£0.08			
FM economy	-£0.06	-£0.02	£0.00	£0.02	£0.03	£0.04	£0.06	£0.08	£0.08			
FM value	-£0.08	-£0.08	-£0.08	-£0.08	-£0.08	-£0.07	-£0.07	-£0.07	-£0.07			
FM sub value	-£0.08	-£0.07	-£0.06	-£0.05	-£0.05	-£0.02	£0.01	£0.03	£0.04			
RYO premium	-£0.03	-£0.02	-£0.02	-£0.02	-£0.01	£0.00	£0.04	£0.07	£0.08			
RYO mid-price	-£0.04	-£0.03	-£0.02	-£0.01	-£0.01	£0.02	£0.04	£0.04	£0.06			
RYO value	-£0.05	-£0.06	-£0.05	-£0.04	-£0.05	-£0.04	-£0.03	-£0.02	-£0.02			

* 2009 begins in May because the budget was in April rather than March and 2015 ends in December because this was the end of the data series

** FM premium and FM mid-price are 20 stick packs, FM value and FM sub value are 19 stick packs (except FM value in 2009 are 20 sticks because 19 sticks were not available), RYO is 12.5g packs.

References

1. Lane Keller K. Designing and implementing brand architecture strategies. *Journal of Brand Management* 2014;21(9):702-15. doi: 10.1057/bm.2014.38
2. Dewhirst T. Into the black: Marlboro brand architecture, packaging and marketing communication of relative harm. *Tob Control* 2017
3. Gallus S, Lugo A, La Vecchia C, et al. Pricing Policies And Control of Tobacco in Europe (PPACTE) project: cross-national comparison of smoking prevalence in 18 European countries. *European journal of cancer prevention : the official journal of the European Cancer Prevention Organisation (ECP)* 2014;23(3):177-85. doi: 10.1097/cej.0000000000000009 [published Online First: 2014/01/21]
4. Scottish Grocer. Tobacco: still searching for good value. *Scottish Grocer* 2014 June. http://www.scottishgrocer.co.uk/2014/06/still-searching-for-good-value/.
5. HMRC. HM Revenue and Customs Value Added Tax (VAT) Bulletin - July 2016, 2016.
6. HMRC. HM Revenue and Customs Tobacco Bulletin - July 2016, 2016.
7. HMRC. Cigarettes: 'Flash' marks and promotional material: significance for duty calculation. *HMRC internal manual tobacco products duty* 2016 16th August; (TPD7070). https://www.gov.uk/hmrc-internal-manuals/tobacco-products-duty/tpd7070.
8. HMRC. Cigarettes meaning of 'description' or 'brand' for duty purposes. *HMRC internal manual tobacco products duty* 2016 16th August; (TPD7060). https://www.gov.uk/hmrc-internal-manuals/tobacco-products-duty/tpd7060.
